# Highly Similar Tetramerization Domains from the p53 Protein of Different Mammalian Species Possess Varying Biophysical, Functional and Structural Properties

**DOI:** 10.3390/ijms242316620

**Published:** 2023-11-22

**Authors:** Shuya Sakaguchi, Natsumi Nakagawa, Haytham M. Wahba, Junya Wada, Rui Kamada, James G. Omichinski, Kazuyasu Sakaguchi

**Affiliations:** 1 Department of Chemistry, Faculty of Science, Hokkaido University, Sapporo 060-0810, Japan; shuya@sci.hokudai.ac.jp (S.S.); n-nakagawa@sci.hokudai.ac.jp (N.N.); junya.wada@nih.gov (J.W.); kamadar@sci.hokudai.ac.jp (R.K.); 2 Département de Biochimie et Médicine Moléculaire, Université de Montréal, C.P. 6128 Succursale Centre-Ville, Montréal, QC H3C 3J7, Canada; haytham.wahba@gmail.com; 3 Department of Biochemistry, Faculty of Pharmacy, Beni-Suef University, Beni-Suef 2722165, Egypt

**Keywords:** evolution, hydrophobic core, Met oxidation, tetramerization, thermal stability, tumor suppressor protein p53

## Abstract

The p53 protein is a transcriptional regulatory factor and many of its functions require that it forms a tetrameric structure. Although the tetramerization domain of mammalian p53 proteins (p53TD) share significant sequence similarities, it was recently shown that the tree shrew p53TD is considerably more thermostable than the human p53TD. To determine whether other mammalian species display differences in this domain, we used biophysical, functional, and structural studies to compare the properties of the p53TDs from six mammalian model organisms (human, tree shrew, guinea pig, Chinese hamster, sheep, and opossum). The results indicate that the p53TD from the opossum and tree shrew are significantly more stable than the human p53TD, and there is a correlation between the thermostability of the p53TDs and their ability to activate transcription. Structural analysis of the tree shrew and opossum p53TDs indicated that amino acid substitutions within two distinct regions of their p53TDs can dramatically alter hydrophobic packing of the tetramer, and in particular substitutions at positions corresponding to F341 and Q354 of the human p53TD. Together, the results suggest that subtle changes in the sequence of the p53TD can dramatically alter the stability, and potentially lead to important changes in the functional activity, of the p53 protein.

## 1. Introduction

The p53 protein is the founding member of a family of transcriptional regulatory proteins that also includes the related factors p63 and p73 [1,2,3,4]. In humans, the p53 protein functions as an important tumor suppressor through its role in controlling key cellular processes associated with DNA repair and genome maintenance [5,6,7]. Significantly, mutations in the p53 gene have been shown to be directly linked to approximately 50% of all known human cancers [8,9,10]. The human p53 protein contains 393 amino acids and it can be roughly divided into four key functional domains. The four domains include a central core DNA-binding domain [11], an N-terminal transcriptional activation domain [12], a tetramerization domain (TD) [8] located just C-terminal to the DNA-binding domain, and a C-terminal regulatory domain that is controlled through numerous post-translational modifications [13,14,15,16].

To bind to a specific DNA target site and function as a transcriptional regulatory factor, the human p53 protein must first form a homo-tetrameric structure [17]. Formation of the tetrameric structure occurs in response to increases in cellular levels of p53 following the activation of its expression, and the activation of p53 expression occurs in response to a variety of external stimuli including oxidative stress and DNA damage [18,19,20]. Importantly, the stability of the tetrameric form of p53 appears to correlate with its transcriptional activity [21,22]. The formation of the p53 tetrameric structure requires a short TD region, which is located between residues 324 and 356 in the sequence of human p53 (HU-p53TD). The structure of HU-p53TD has been determined by both NMR spectroscopy and X-ray crystallography, and it consists of four monomers that each contain a single β-strand (residues 326–333) followed by a single α-helix (335–356), which are separated by a tight turn of Gly334 [23,24]. To generate the tetrameric form of p53, two monomers form an initial dimeric structure through interactions between their β-strands that results in a two-stranded β-sheet as well as hydrophobic packing between the helices from each monomer. Next, two dimers come together to form the tetramer, which is stabilized by a helical bundle that forms through a series of hydrophobic and electrostatic contacts between the side chains of the amino acids located in the four α-helices from each monomer.

Although all members of the p53 family of proteins bind to their target DNA sequences as tetramers, the structure of HU-p53TD is distinct from the structure of both HU-p63TD and HU-p73TD. The TDs of human p63 and p73 both contain an additional helical segment, which is located at the C-terminal end of the TD, creating a βαα motif in comparison to the more simplistic βα motif found in HU-p53TD [25,26]. In the case of p73, the second helical domain has been shown to not only increase the stability of the homo-tetramer, but also to play a crucial role in the formation of hetero-tetramers between the human p63 and p73 proteins. In contrast, the formation of hetero-tetramers between the human p53 protein and either p63 or p73 has not been observed, and this inability of p53 to form hetero-tetramers has been attributed to the absence of the second helical segment in HU-p53TD [27]. Interestingly, this second helical region has been shown to be present in the TD of the Zebrafish p53 (ZF-p53TD), which is from a subgroup of bony fishes; however, to date, there is no evidence that ZF-p53TD forms hetero-tetramers with either its p63 or its p73 [28]. The presence of these structural variations in p53TD between different species suggests that the TD of p53 may have evolved so that it could perform different functions in the transition from aquatic species to mammalian terrestrial-based species [29,30,31].

The common tree shrew is a small rodent-like animal that has drawn considerable scientific interest since it possesses numerous characteristics in common with primitive primate species. Although the tree shrew is estimated to have diverged from larger primates about 90 million years ago, it is still regarded as a potential model for examining early primate evolution since it represents an early precursor in the evolution pathway leading to apes and humans [32,33,34]. Surprisingly, we recently determined that the TD of the tree shrew p53 protein (TS-p53TD) is dramatically more thermostable than HU-p53TD despite the fact that the two TDs share very a high sequence identity percentage [35]. It was postulated that this difference was important in helping the tree shrew adapt to its unusual diet that includes the daily consumption of large quantities of alcohol, which poses an enhanced risk of DNA damage due to exposure to high levels of the genotoxic agent acetaldehyde. This increase in the thermostability of the TS-p53TD also suggests that, within different mammalian species, the p53 proteins may have evolved to serve different functional roles by altering the biophysical properties of their TD, which can be produced through minimal alterations in the sequence.

To determine whether the p53TDs from different mammalian species have distinct biophysical properties despite sharing high sequence identity percentages, we examined the secondary structure, the thermostability, the transcriptional activity, and several of the structures of the p53TDs from six different mammalian species (human, tree shrew, guinea pig, Chinese hamster, sheep, and opossum). These mammalian species were selected because they represent important model systems in scientific research. Our results demonstrate that, despite sharing very high sequence identity percentages and similar overall three-dimensional structures, there are significant differences in the thermostability of the different p53TDs, with the opossum and tree shrew showing the largest enhanced thermostability relative to the HU-p53TD. This enhanced thermostability appears to correlate with an increase in transcriptional activity of the proteins, and, in the case of the tree shrew p53TD, with slower rates of oxidation of a methionine residue within its hydrophobic core. Structural characterization of the p53TD from tree shrew and opossum highlights how even subtle changes in the sequence can result in a more compact organization of the hydrophobic core formed between the four monomers within the tetrameric structure to enhance the thermostability. Together, these results further demonstrate how substitutions within the sequence of mammalian p53TDs can alter their properties, and suggest that simple changes may be associated to important functional differences in the p53 protein in the different species as well as to cancer-associated mutations in humans.

## 2. Results

### 2.1. Selection and Preparations of TDs from Mammalian p53 Protein

**Figure 1:**
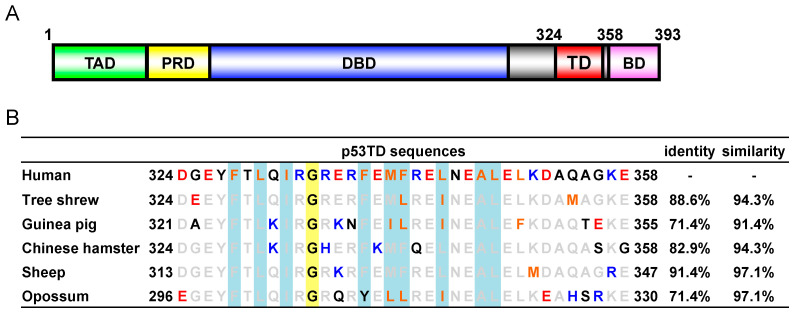
Mammalian p53TDs. (**A**) Schematic structure of the human p53 protein. TAD: transactivation domain, PRD: proline-rich domain, DBD: DNA binding domain, TD: tetramerization domain, BD: basic domain. (**B**) Sequences of the mammalian p53TDs and % of identity/similarity relative to the HU-p53TD. Conserved residues in terms of human are gray, substituted residues are hydrophobic, orange; acidic, red; basic, blue; others, black. Residues forming a hydrophobic core are shadowed in light blue.

Six mammalian species (human, tree shrew, guinea pig, Chinese hamster, sheep, and opossum) were chosen for testing of the biophysical and functional properties of their p53TD, because they each represent an important mammalian model species [36,37,38,39,40,41,42]. The amino acid sequence selected as the TD for each species was chosen based on sequence alignment to the well-characterized HU-p53TD, which corresponds to residues 324–358 of the human p53 protein (Figure 1).

Thus, the TDs from each of the six species contain 35 amino acids. Peptides containing the sequences of the six different p53TDs were initially prepared by solid-phase peptide synthesis using Fmoc-chemistry. Comparing the sequences of the p53TDs from the six species shows (Figure 1) that the percentages of sequence identity of the five other mammalian TD sequences relative to the HU-p53TD range from a high of 91.4% for the Sheep p53TD (SH-p53TD) to a low of 71.4% for both the Opossum p53TD (OP-p53TD) and the Guinea pig p53TD (GP-p53TD). In addition, the five sequences shared more than 90% similarity with the HU-p53TD, with a high of 97.1 for the SH-p53TD and OP-p53TD, whereas the low was 91.4% for the GP-p53TD. Overall, the sequences for the p53TDs from the different mammalian species are very similar.

### 2.2. Secondary Structure Composition of the Six p53TD Based on Circular Dichroism (CD) Analysis

**Figure 2:**
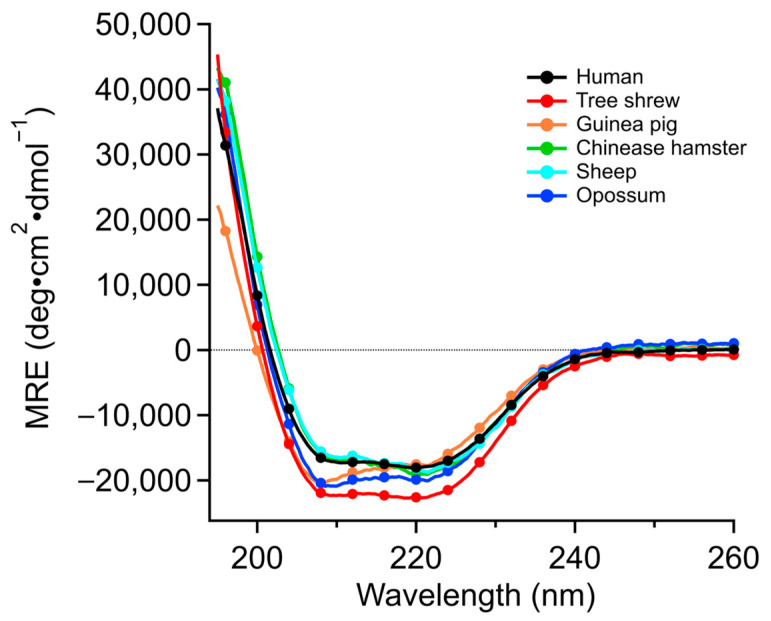
CD spectra of the mammalian p53TDs. The secondary structures of the mammals’ p53TDs were measured by CD spectrometry at 4 °C.

To initially characterize their structural characteristics, the secondary structure compositions of the six different mammalian p53TDs were determined using circular dichroism (CD) spectroscopy. Based on the previous structures of the HU-p53TD, the peptides should theoretically contain 63% helix, 23% beta, 3% turn, and 11% random structural content (Table 1). Analysis of the six p53TDs by CD spectroscopy in solution indicated that the OP-p53TD and TS-p53TD give the closest matches in terms of helical structure to the theoretical value of 63%, with predicted helical contents of 62% and 64%, respectively, based on CD analysis (Figure 2 and Table 1).

In terms of beta content, the GP-p53TD and the TS-p53TD were the closest matches to the theoretical value of 23%, with both containing 24% beta content as estimated by CD analysis (Table 1). Overall, the secondary structure content of the TS-p53TD in solution based on the CD analysis was the closest to the theoretical values based on the crystal structure of the HU-p53TD. Although NMR measurements have demonstrated that the HU-p53TD is dynamically stable in solution between residues 325 and 347, the differences between the theoretical values based on the reported structure [23] and the measured values for the HU-p53TD determined by CD analysis could represent the relative association and disassociation of the HU-p53TD that occurs in solution.

### 2.3. Verification of Tetrameric Structure of the Mammalian p53TDs by Gel Filtration Chromatography

**Figure 3:**
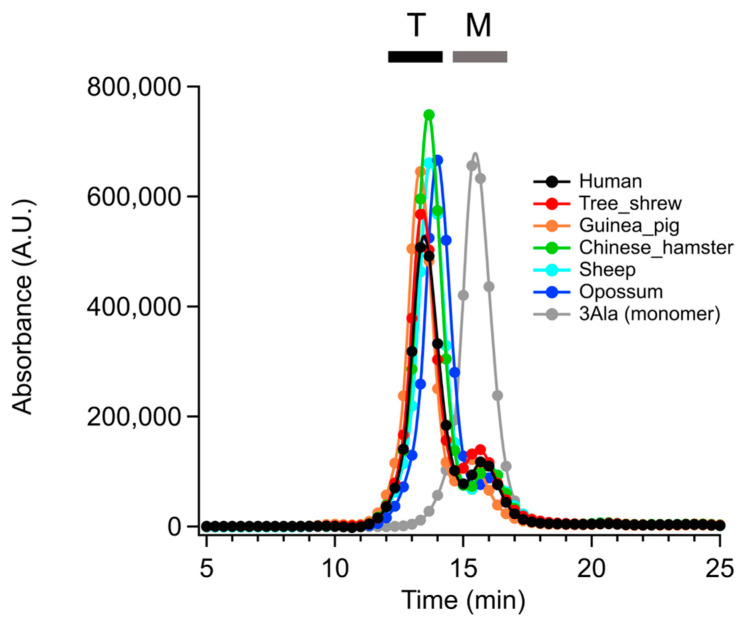
Gel filtration of mammals’ p53TD. The oligomeric states of the mammalian p53TDs and 3Ala (monomer mutant) were measured by gel filtration chromatography. The black and gray bar represented the tetramer (T) and monomer (M) fraction, respectively [21]. The UV absorbance was monitored at 214 nm.

To verify that they form tetrameric structures, the six mammalian p53TDs were analyzed by gel filtration chromatography. Using this methodology, we previously demonstrated that the HU-p53TD and the TS-p53TD exist predominantly in the tetrameric form at a concentration of 100 mM in 50 mM phosphate buffer with a pH of 7.5 and with 100 mM NaCl. Using identical experimental conditions, the SH-p53TD, GP-p53TD, OP-p53TD, and CH-p53TD are also all predominantly in their tetrameric form (Figure 3).

The differences between the monomeric and tetrameric forms can be distinguished in this experiment by comparing them with the retention time by gel filtration chromatography for a triple-alanine substituted variant of the HU-p53TD (3Ala), which has been shown to only exist in a monomeric form (Figure 3; grey line). All six of the mammalian p53TDs are predominantly in their tetrameric form under the experimental conditions used.

### 2.4. Determination of the Thermostability of Mammalian p53TDs by CD Spectroscopy

**Figure 4:**
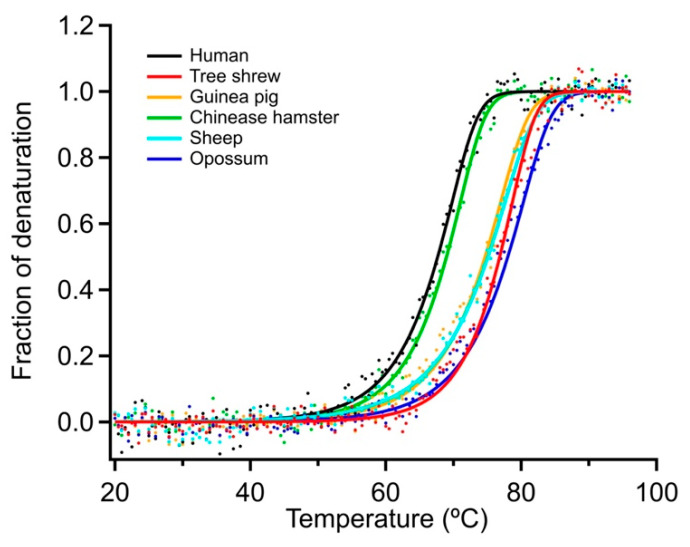
Thermal denaturation curves of the mammalian p53TDs. The signals from the mammalian p53TDs were monitored by CD spectrometry at 222 nm between 4 °C and 96 °C. The fraction of denaturation at each temperature was plotted to generate the denaturation curves.

Given that the TS-p53TD was previously shown to have much higher thermostability than the HU-p53TD despite the two sharing a very high sequence identity percentage, we measured the thermostability of the other four mammalian p53TDs based on CD measurements at variable temperatures. In these experiments, the ellipticity of each p53TD at 222 nm was measured between 4 °C and 96 °C, and these measurements were used to generate denaturation curves for each peptide (Figure 4). The different denaturation curves were then used to calculate the *T*_m_, the *ΔT*_m_, and the *ΔG*_u_^37°C^ for each of the six mammalian p53TDs (Table 2). As seen with the TS-p53TD, all of the other mammalian p53TDs are significantly more stable than the HU-p53TD, with the exception of the CH-p53TD. Of the six mammalian p53TDs tested, the OP-p53TD is the most thermostable. In the case of the OP-p53TD, the calculated *T*_m_ was 78.0 °C and the *ΔG*_u_^37°C^ was 36.6 kcal/mol, which is very similar to the TS-p53TD, which has a *T*_m_ of 77.1 °C and a *ΔG*_u_^37°C^ of 36.4 kcal/mol, but significantly higher than the HU-p53TD’s calculated *T*_m_ of 67.8 °C and *ΔG*_u_^37°C^ of 32.4 kcal/mol. In addition to the dramatic increase in thermostability observed for the OP-p53TD and TS-p53TD, the SH-p53TD and the GP-p53TD showed an intermediate increase in thermostability relative to the HU-p53TD and CH-p53TD. For the SH-p53TD, the calculated *T*_m_ was 75.1 °C and the *ΔG*_u_^37°C^ was 34.0 kcal/mol, and for the GP-p53TD, the calculated *T*_m_ was 74.7 °C and the *ΔG*_u_^37°C^ was 35.4 kcal/mol. Taken together, these results suggest that there are considerable differences in the thermostabilities of the p53TDs from the six different mammalian species despite the fact that their sequences are often quite similar.

### 2.5. Transcriptional Activity of Chimeric p53 Protein with TD of Each Species

**Figure 5:**
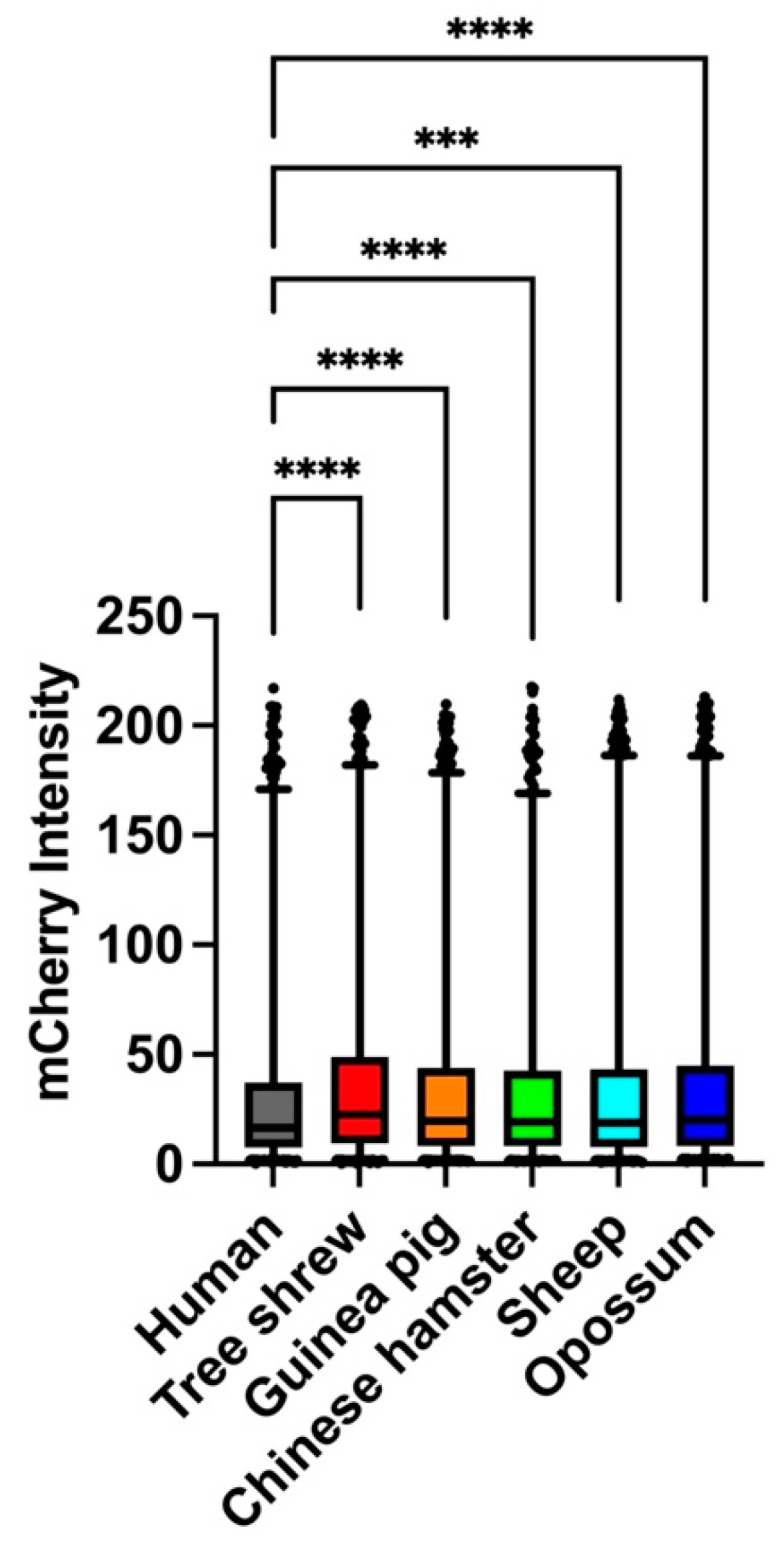
Transcriptional activity of the chimeric human p53 protein substituted with the TDs from the other mammalian p53 proteins in single cells. The proteins were expressed at the physiological protein level corresponding to levels of the human p53 in cells. The mCherry (p53-dependent transcription) fluorescence signals in each single cell were quantified. Boxes and whiskers include the values between the 25th and 75th, and 1st and 99th, percentiles, respectively. More than 3000 cells were analyzed under each condition for three independent experiments. Significance was analyzed by using Kruskal–Wallis test. *** *p* < 0.0005, **** *p* < 0.0001.

It has been suggested that the transcriptional activity of the p53 protein is correlated with the stability of its tetrameric structure. In order to test whether changes in the thermostability of the different mammalian p53TDs alter their relative transcriptional activity, we generated expression vectors to produce chimeric p53 proteins in which the TD from each of the five other mammalian species was substituted into the corresponding position in the human p53 with p53TD, and the transcriptional activity of each chimeric p53 protein was measured using a reporter assay (Figure 5 and Figure S1). The transcriptional activities of several of the chimeric p53 proteins were slightly but significantly increased for several species in comparison with the wild-type human p53 protein. Among the different mammalian species, the chimeric p53 containing either the OP-p53TD or the TS-p53TD showed the highest transcriptional activity towards the p21 promoter used in this assay, suggesting that enhanced thermostability of the p53TD increases the transcriptional activity under these experimental conditions. Overall, these results were consistent with our previous data which indicated that the transcriptional activity of p53 was associated with the thermostability of p53TD [21,22].

### 2.6. Crystal Structures of HU-p53TD, TS-p53TD and OP-p53TD

**Figure 6:**
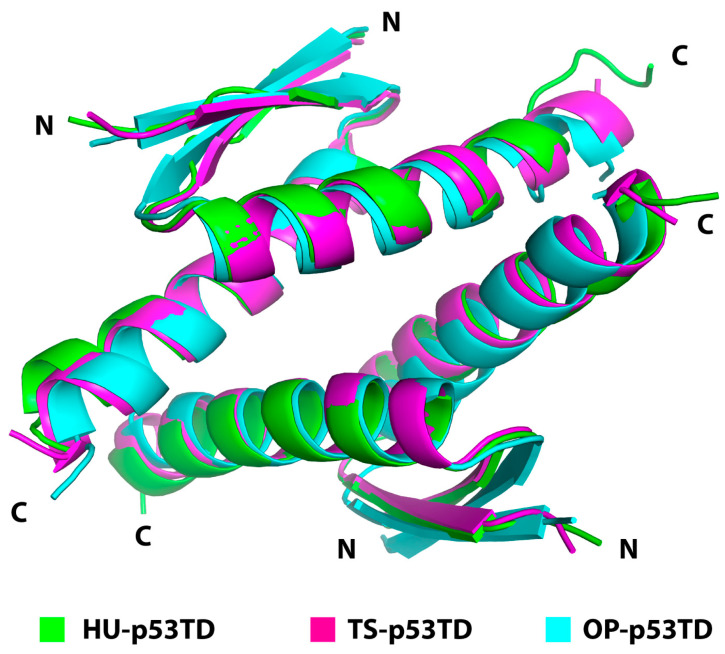
Overlay of the crystal structures of HU-p53TD (Green), TS-p53TD (Magenta), and OP-p53TD (Aqua). The structures are shown as cartoon representations and are overlayed based on the C*α* positions of the peptide backbone. The N-terminal (N) and the C-terminal (C) of each monomer is indicated for clarity. The root mean square deviation (r.m.s.d.) for the Cα positions of the peptide backbone are 0.39 Å between the HU-p53TD and the TS-p53TD, 0.95 Å between the HU-p53TD and the OP-p53TD, and 0.75 Å between the TS-p53TD and the OP-p53TD.

To help define the differences between the HU-p53TD and the mammalian p53TDs with significantly enhanced thermostability at the atomic level, we generated crystals of the OP-p53TD and the TS-p53TD as well as the HU-p53TD using the identical sequences employed in the other assays. After screening a variety of different conditions, crystals were obtained for the HU-p53TD that diffracted to a resolution of 1.1 Å, for the TS-p53TD that diffracted to a resolution of 1.17 Å, and for the OP-p53TD that diffracted to a resolution of 1.35 Å (Table S2). From these crystals, we were able to obtain the first high-resolution structures for the TS-p53TD and the OP-p53TD as well as a very high-resolution structure for the HU-p53TD. As expected, the structures of the three p53TDs are very similar, in that they consist of a tetramer that is composed of two dimers and each monomer contains a βα fold (Figure 6).

### 2.7. Comparison of the Structure of the TS-p53TD with the HU-p53TD

**Figure 7:**
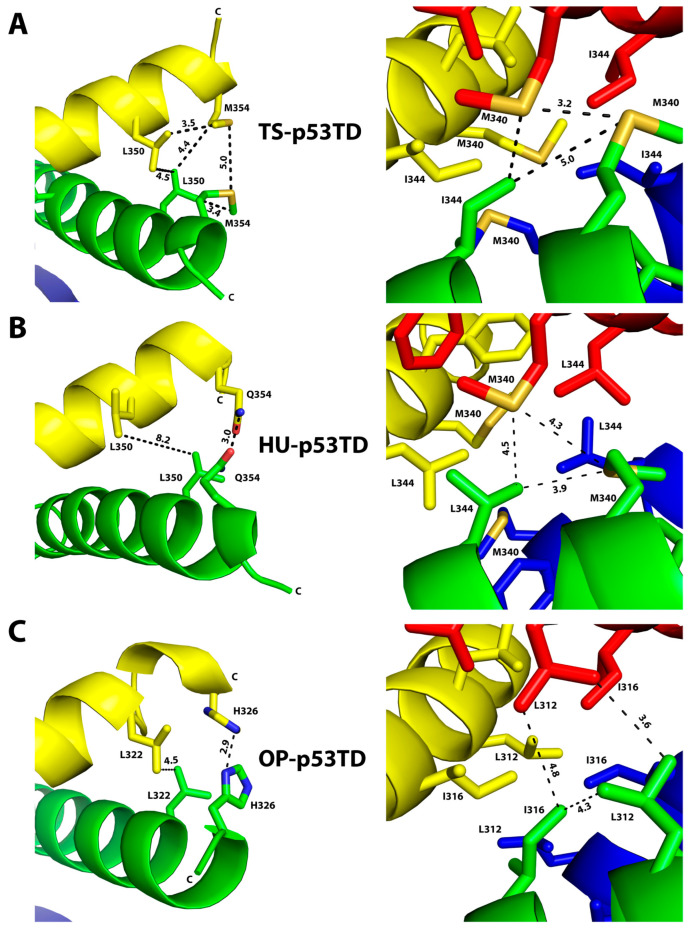
Key differences in the dimeric and tetrameric interfaces of the TS-p53TD and OP-p53TD that enhance their thermostability in comparison to the HU-p53TD: Close up and metrics (dashed distance lines between residues in Å) of the TS-p53TD (**A**), HU-p53TD (**B**), and OP-p53TD (**C**), highlighting the differences in the C-terminal regions of dimers (**left panel**) and the hydrophobic core of the tetrameric interfaces (**right panel**). The three structures are shown as cartoons with each monomer in a unique color (red, blue, yellow, and green) and the interacting amino acids associated with the key differences shown as stick conformations. The N-terminal (N) and the C-terminal (C) of each monomer is indicated for clarity when possible, and the side chains are color-coded based on the atom type (S-yellow, O-red, and N-blue).

In comparing the TS-p53TD with the HU-p53TD structure, there are two major regions where structural differences are observed, and these differences occur in areas that contain the three amino acid differences in the two sequences within the structured region of these p53TDs. The first difference occurs in the region around L350 and Q354 in the HU-p53TD, which corresponds to L350 and M354 in the TS-p53TD (Figure 7 and Figure S4). In the structure of the TS-p53TD (Figure 7A), the side chains of L350 and M354 from the adjacent monomers are in proximity to each other and in position to make hydrophobic contacts with the same residues of the other monomer within the dimer (Figure 7A, left panel and Figure S4, upper panel). This results in the formation of two hydrophobic clusters, one at the C-terminal end of the helices in each dimer. In contrast, in the HU-p53TD (Figure 7B), the side chain of Q354 from one monomer forms a hydrogen bond with the second Q354 of the adjacent monomer in the dimers in the HU-p53TD (Figure 7B, left panel and Figure S4, upper panel); however, the two L350 residues from the adjacent monomers remain more distant from each other than the corresponding residues in the TS-p53TD (4.5 versus 8.2 Å). The second key difference between the TS-p53TD and HU-p53TD structures occurs in the hydrophobic core, formed between the four helices, that stabilizes the tetrameric structure. In this region, the aromatic side chain of F341 in the HU-p53TD is substituted by the aliphatic L341 in the TS-p53TD and L344 in the HU-p53TD is substituted by I344 in the TS-p53TD. This substitution at position 341 leads to a series of subtle difference in the packing of the side chains that form the hydrophobic core between the four helices. It is noteworthy that the distances between the sulfur atoms of the M340 residues from the different dimers are 1.1 Å closer (3.2 versus 4.3 Å) to each other in the TS-p53TD structure (Figure 7A, right panel and Figure S4, lower panel) than in the HU-p53TD structure (Figure 7B, right panel and Figure S4, lower panel). In addition, the sulfur atom of M340 from one dimer is 0.5 Å closer (4.0 versus 4.5 Å) to the nearest methyl group of I344 of the other dimer in the TS-p53TD (Figure 7A, right panel) than the sulfur atom of M340 is to the nearest methyl group of L344 of the other dimer in the HU-p53TD (Figure 7B, left panel). Together, these two differences help explain the enhanced thermostability of the TS-p53TD in comparison to the HU-p53TD.

### 2.8. Comparison of the Structure of the OP-p53TD with the HU-p53TD

As seen with the TS-p53TD, there are also two major structural differences observed in comparing the structure of the OP-p53TD (Figure 7C and Figure S5) with the structure of the HU-p53TD (Figure 7B and Figure S5), and these differences occur in the same two regions. In the first region, L350 and Q354 in the HU-p53TD correspond to L322 and H326 in the OP-p53TD, and in the structure of the OP-p53TD (Figure 7C, left panel and Figure S5, upper panel), the imidazole rings from the side chains of the H326 residues from the adjacent monomers are in proximity (2.9 Å) to form a hydrogen bond between each other. As seen with the TS-p53TD (Figure 7A, left panel and Figure S5, upper panel), this helps stabilize a hydrophobic interaction by bringing the side chains of the L322 residues from the adjacent monomers in proximity to each other (4.5 Å in the OP-p53TD versus 8.2 Å in the HU-p53TD) [43]. The second key difference between the OP-p53TD and HU-p53TD structures occurs in the hydrophobic core formed between the four helices. In this region, as seen with the TS-p53TD, the aromatic side chain of F341 in the HU-p53TD is substituted by the aliphatic L313 in the OP-p53TD and L344 in the HU-p53TD is substituted by I316 in the OP-p53TD. In addition, M340 of the HU-p53TD is replaced by L312 in the OP-p53TD (Figure 7C, right panel and Figure S5, lower panel). As seen with the TS-p53TD (Figure 7A, right panel and Figure S5, lower panel), this substitution at position 341 leads to a series of subtle difference in the packing of the side chains that form the hydrophobic core between the four helices in comparison to what is observed for the HU-p53TD (Figure 7B; right panel and Figure S5; lower panel). Together, these two differences not only help explain the enhanced thermostability of the OP-p53TD in comparison to the HU-p53TD, but also explain why its thermostability is similar to what is observed for the TS-p53TD.

### 2.9. Oxidation of Methionine Residues in HU-p53TD and TS-p53TD

**Figure 8:**
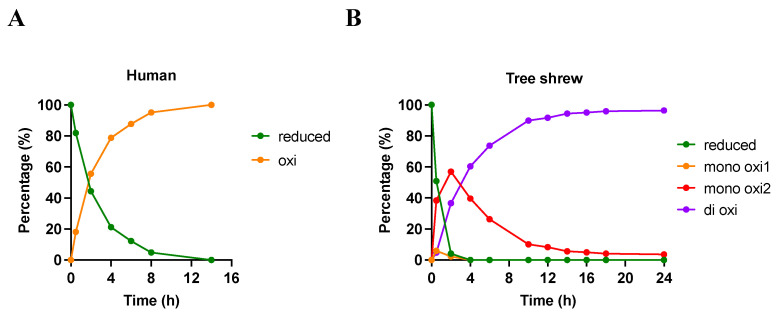
Oxidation of the mammalian p53TDs (**A**: HU-p53TD, **B**: TS-p53TD) by hydrogen peroxide. The peptide solution was incubated in the presence of hydrogen peroxide. The reaction mixture of each indicated time point was analyzed by HPLC and the observed peak areas corresponding to each oxidation status, which were determined by MALDI-TOF MS (Table 3), were plotted.

The HU-p53TD contains one Met residue (M340) in its hydrophobic core, whereas the TS-p53TD contains two Met residues (M340 and M354), one in the hydrophobic core and one near the C-terminal interface of the helices. Given that the M354 of the TS-p53TD is the key determinant of its enhanced stability relative to the HU-p53TD and that M340 in the HU-p53TD appears to be more surface-exposed than the M340 of the TS-p53TD, we evaluated the relative accessibility of these three Met residues to oxidation by hydrogen peroxide (H_2_O_2_). The rate of oxidation of the Met residues was assessed using an HPLC assay in combination with mass spectral (MS) analysis (Figure 8 and Table 3).

In the presence of H_2_O_2_, the M340 of the HU-p53TD oxidized in a time-dependent manner and one additional peak (oxi) appeared in the HPLC chromatogram due to oxidation of the M340. After 2 h, more than half of the M340 was in the oxidized form, and after 8 h, more than 95% was oxidized. By 14 h, the HU-p53TD was completely in an oxidized form. In contrast, three new peaks appeared in the HPLC chromatogram following the addition of H_2_O_2_ to a solution containing the TS-p53TD. The MS analysis demonstrated that two of these peaks were the peptide in which either M354 (mono-oxi1) or M340 (mono-oxi2) was oxidized, and the other peak corresponded to the peptide in which both Met residues were oxidized (di oxi). The level of the non-oxidized TS-p53TD was less than 5% of the total amount after 2 h. The M354 oxidized peak was present only at 30 min and 2 h after the initiation of the oxidation reaction, whereas the M340 oxidized peak increased over the first 2 h and then began to decrease. The di-oxidized peak gradually increased in a time-dependent manner and exceeded 95% at 16 h after the initiation of the oxidation reaction.

To identify the oxidized products, each peak from the HPLC separation of the two reactions was collected and treated with trypsin (Table 3). MS analysis of peptide fragments from the different peaks following the trypsin treatment revealed that the mono-oxi1 peak corresponds to the oxidation of M340 in both the HU-p53TD and TS-p53TD samples. This result indicates that the mono-oxi2 peak from the TS-p53TD sample corresponded to the oxidation of M354, and this indicates that M354 is more easily oxidized than M340 in the TS-p53TD. In addition, the results demonstrate that M340 in the TS-p53TD oxidizes at a slower rate than the M340 of the HU-p53TD under these conditions, which we postulate is due to the increased stability of the TS-p53TD relative to the HU-p53TD.

## 3. Discussion

Amongst the different mammalian species analyzed in this study, the opossum, as a member of the marsupial family, is the most evolutionarily ancient species, whereas the tree shrew, as a member of the Scandentia family, is evolutionarily the closest to humans. Despite these two species being the farthest and nearest to humans in terms of evolution, the TDs from these two species displayed the most significant enhancements in thermostability and ability to activate transcription relative to that of the HU-p53TD. Based on the crystal structures of the OP-p53TD and TS-p53TD, the structural changes that lead to their enhanced thermostability appear to be centered in the same two regions of their sequence. The first region is centered around the C-terminal of the α-helix and this leads to an enhanced stabilization of the dimers, and the second region is centered around residues in the central area of the α-helix that form the hydrophobic core to stabilize the tetrameric structure. These two major differences are consistent with the fact that, in the amino acid sequences of the different mammalian p53TDs, the sequences of the α-helical region are considerably more diversified than the sequences of the more highly conserved β-sheet region.

Our analysis of the crystal structures of the TS-p53TD and the OP-p53TD indicates that the key residues in the C-terminal region that help to enhance the stability of the dimers are M354 in the TS-p53TD and H326 in the OP-p53TD, which corresponds to Q354 in the HU-p53TD. In both the TS-p53TD and OP-p53TD, the side chains of M354 or H326 form key interactions with themselves as well as with a neighboring Leu residue (L350 and L332 in tree shrew and opossum, respectively) located four residues in front of them in their sequences. This enables that Leu residue from one monomer to from hydrophobic contacts with the same Leu residue on the other monomer of the dimer. In the HU-p53TD, Q354 is not able to stabilize the hydrophobic contacts between the two Leu350 residues of the dimer and this results in a decrease in the thermostability of the HU-p53TD relative to the TS-p53TD and OP-p53TD. In the central region of the α-helix, the hydrophobic core is formed by the side chains of the residues F328, L330, I332, F338, M340, F341, L344, A347, and L348 [44,45]. Within these residues that make up the hydrophobic core, M340, F341, and L344 in the HU-p53TD appear to be more commonly substituted for other amino acids in the sequences of other mammalian p53TDs. These three residues in the HU-p53TD correspond to L312, L313, and I317 in the OP-p53TD, and M340, L341, and I344 in the TS-p53TD, respectively. In comparing the three sequences, the biggest difference appears to be the presence of the aromatic F341 in the HU-p53TD relative to the aliphatic Leu residue in the OP-p53TD (L313) and the TS-p53TD (L341). Our analysis of the three crystal structures showed that the OP-p53TD and TS-p53TD form a more compact hydrophobic core relative to what is observed for the HU-p53TD. In the TS-p53TD, this results in the sulfur atoms of M340 being closer to each other as well as to the nearest methyl group of I344 than they are in the HU-p53TD. This more compact hydrophobic core is also supported by the fact that the oxidation rate of M340 was slower for the TS-p53TD in comparison with the HU-p53TD. Overall, this suggests that replacing the aromatic F341 residue in the hydrophobic core with a hydrophobic aliphatic amino acid increases the stability of the TS-p53TD and OP-p53TD.

The role of aromatic F341 in destabilizing the core of the HU-p53TD relative to the OP-p53TD and TS-p53TD is interesting in light of previous studies examining the role of the three different Phe residues in the stability of the HU-p53TD. In this study, the aliphatic cyclohexylalanine group was substituted in place of F328, F338, and F341 in the HU-p53TD. When this hydrophobic aliphatic group was substituted for either F328 or F338, a decrease in the thermostability of the HU-p53TD was observed, suggesting that it was important to have the aromatic residue in these positions. In contrast, substituting the aliphatic cyclohexylalanine group for F341 of the HU-p53TD lead to a significant enhancement in the thermostability from a *T*_m_ of 67.8 °C to a *T*_m_ of more than 90 °C [46]. These results suggest that the substitution of an aliphatic group at the positions corresponding to the aromatic F328 and F338 of the HU-p53TD significantly decreases the thermostability of the p53TD, but the substitution of the aliphatic hydrophobic group in place of an aromatic F341 of the HU-p53TD leads to a significant enhancement. These results support the conclusion that substitution of the aromatic F341 by an aliphatic hydrophobic amino acid in the TS-p53TD and OP-p53TD is in part responsible for their enhanced thermostability relative to the HU-p53TD.

Based on the conclusions that differences in two regions make important contributions to the overall stability of the p53TD, we examined these regions in the three remaining species evaluated as part of this study. The CH-p53TD sequence contains the same amino acid residues in the positions corresponding to Q354 at the C-terminal as well as M340, F341, and L344 in the center of the α-helix of the HU-p53TD. This is consistent with our results showing that the CH-p53TD has nearly the same thermostability as the HU-p53TD. In contrast, the GP-p53TD has three aliphatic residues (Ile, Leu, and Leu) in the central region of the α-helix, like the TS-p53TD and the OP-p53TD, but a Gln residue in the position of Q354 in the C-terminal helical region, like the HU-p53TD. Thus, there should be enhanced stability in the core region of the tetramer due to the replacement of the aromatic F341 residue, but no enhanced stability in the C-terminal region of the helix relative to the HU-p53TD. Again, this is consistent with our results which demonstrated that the GP-p53TD has an intermediate thermostability between what is observed for the HU-p53TD and what is observed for the TS-p53TD and OP-p53TD. The last sequence to compare is that of the SH-p53TD, which is the same as the HU-p53TD and CH-p53TD in both regions. However, the SH-p53TD possesses an intermediate thermostability relative to the HU-p53TD and the TS-p53TD/OP-p53TD. Surprisingly, the HU-p53TD and SH-p53TD share 91.4% of their sequence identities and the only two differences occur at E336 and K357 of the HU-p53TD, which correspond to K325 and R346, respectively, in the SH-p53TD. Given that the K357/R346 residue is outside of the structured region of the p53TD, this indicates that E-to-K substitution at position 336 in the HU-p53TD (position 325 in SH-p53TD) must be responsible for the intermediate thermostability observed for the SH-p53TD.

In conclusion, the results demonstrate that the biophysical properties of the different mammalian p53TDs can be altered fairly dramatically by rather subtle changes in the amino acid sequence, and this could lead to important functional differences for their respective p53 proteins. In tree shrew, the average body temperature is approximately the same as for humans, but the circadian range of body temperature differs by two-fold [47]. Mitochondrial UCP1-mediated thermogenesis is important for regulating body temperature, and radical oxygen species (ROSs) are produced during this process [48]. This suggests that tree shrews are more likely to be exposed to ROS-mediated oxidative stress than humans due to heat production associated with circadian fluctuations. To increase its resistance to ROS-induced oxidative stress, the TS-p53TD appears to have undergone a structural evolution to become more thermostable, which may be advantageous to its survival through changes in the activity of its p53 proteins. In the case of opossums, they are marsupial, and unlike humans and other placentals, they have their offspring immature, which grow in their pouches by feeding on their mother’s milk. Since these immature progenies are exposed to various stresses early in life, including UV radiation [42,49], an enhanced stability of the p53 protein may have been required for a more optimal tumor suppressor function. In humans, a number of tumors have been reported that contain single point missense mutations within the p53TD. In many cases, these point mutations lead to the substitution of an amino acid residue in the hydrophobic core of the tetramer, which results in the destabilization of the tetrameric structure and, in many cases, the complete loss of the proteins’ functions [21,42]. However, our results indicate that mutations that lead to subtle amino acid substitutions in the p53TD could also lead to an enhancement in the overall stability and transcriptional activity of the p53 protein. This could result in an increase in function that could either be beneficial or detrimental to the organism. Future studies will be needed to identify mutations within the p53TD that may have resulted in enhanced activity of the p53 protein as opposed to mutations that result in the loss of activity.

## 4. Materials and Methods

### 4.1. Synthesis of p53TD Peptides

The peptides corresponding to the human p53 TD (HU-p53TD; residues 324–358 of human p53), the tree shrew p53 tetramerization domain (TS-p53TD; residues 324–358 of tree shrew p53), the guinea pig p53 tetramerization domain (GP-p53TD; residues 321–355 of guinea pig p53), the Chinese hamster p53 tetramerization domain (CH-p53TD; residues 324–358 of Chinese hamster p53), the sheep p53 tetramerization domain (SH-p53TD: residues 313–347 of sheep p53), and the opossum p53 tetramerization domain (OP-p53TD; residues 296–330 of opossum p53) were synthesized as previously described [35]. The peptide sequences are shown in Figure 1. The synthesized peptides were deprotected and cleaved from the resin by using Reagent K (TFA:H_2_O:thioanisole:ethanedithiol:phenol = 82.5:5:5:2.5:5). The peptides were all purified to homogeneity using reverse-phase HPLC over a C8 column (Vydac, Hsperia, CA, USA) and the correct molecular weight of each peptide was verified by MALDI-TOF-MS analysis (Figure S2 and Table S1). Peptide concentrations were determined using an extinction coefficient, ε_280_ = 1490 M^−1^ cm^−1^, which corresponds to a single tyrosine present in all of the p53 proteins.

### 4.2. Determination of Oligomeric State

To verify that the p53TD peptides from the different mammalian species form tetrameric structures, the peptides were analyzed by gel filtration chromatography on a Superdex 75 (GE Healthcare, Chicago, IL, USA) column. For this analysis, 20 µL of each peptide at 100 µM concentration in 50 mM phosphate buffer, pH 7.5 with 100 mM NaCl, was injected onto the column and eluted at room temperature using a flow rate of 0.1 mL/min with UV-monitoring at 214 nm.

### 4.3. Determination of Secondary Structure and Thermal Stability Analysis

To determine the secondary structure content of the different p53TD peptides, circular dichroism (CD) spectra at a peptide concentration of 10 µM in a buffer consisting of 50 mM sodium phosphate, pH 7.5 with 100 mM NaCl, were recorded at 4 °C on a Jasco J-805s spectropolarimeter in a 1 mm quartz cell. Scans were recorded continuously over the region between 195 and 260 nm at a rate of 50 nm/min and a bandwidth of 0.2 nm. The percentages of secondary structural elements were calculated using the commercially supplied JASCO JWSSE-480 software 1.53.07 package and fit according to previously published methods for analysis [50]. For the thermal denaturation assay, CD spectra were recorded between 4 °C and 96 °C and the temperature was increased at a fixed rate of 1.0 °C/min. Ellipticity was measured at 222 nm for monitoring the α-helical content of the peptides. The thermodynamic parameters of the peptides were determined as described previously [21]. The theoretical values were calculated using the PDB:3SAK structure [23].

### 4.4. Preparation of Plasmids for Transcriptional Activity Assay

A series of reporter plasmids, p(p53RE(CDKN1A))-mCherry-NLS-hCMV-Venus-chimeric p53, were constructed to test the transcriptional activity of the different mammalian p53TDs. The plasmid carried the p53RE derived from the CDKN1A gene. For each of the species, a chimeric p53 expression plasmid was prepared, in which the TD of the human p53 protein was replaced by the TD from the other mammalian species (tree shrew, guinea pig, Chinese hamster, sheep, and opossum). The cDNA sequences encoding the TD from the different species were inserted into the human p53 sequence in the vector.

### 4.5. Transcriptional Activity Assay

The transcriptional activities of chimeric p53 proteins containing TD sequences from each of the mammalian species were determined as described previously with minor modifications [51]. H1299 p53 null cells (American Type Culture Collection, Manassas, VA, USA) were seeded in a 35 mm-diameter plastic dish (Falcon, Corning, NY, USA) and grown in RPMI-1640 medium (Sigma-Aldrich, St. Louis, MO, USA) with 10% fetal bovine serum (FBS) (Thermo Fisher Scientific, Waltham, MA, USA) and 100 units/mL penicillin and 100 µg/mL streptomycin (Gibco, Thermo Fisher Scientific, Waltham, MA, USA) in 5% CO_2_. After 12 h, cells were washed three times with 1.5 mL PBS. Next, 100 µL Opti-MEM (Thermo Fisher Scientific, Waltham, MA, USA), 2.4 µg plasmid, and 6 µL Lipofectamine 2000 (Thermo Fisher Scientific, Waltham, MA, USA) were mixed and incubated for 20 min at room temperature. Then, 700 µL Opti-MEM was added to the mixture (transfection mixture). The transfection mixture was added to the dish containing the cells and incubated for 1 h at 37 °C. Afterward, the transfection mixture was removed, and the cells were grown in 1.5 mL of normal culture medium at 37 °C. After 11 h, the cells were washed three times with 900 µL PBS and fixed with 900 µL of a 10% neutral formalin buffer solution (Wako, Osaka, Japan) for 10 min at room temperature with agitation. The formalin solution was removed, and the cells were incubated in 900 µL 0.2% Triton-X-100/PBS for 5 min at room temperature. The cells were washed three times with 900 µL PBS and incubated for 15 min in 1 mL 0.1% DAPI/PBS at room temperature to stain the nuclei. The cells were then washed twice with 900 µL PBS and 1 mL of PBS was added to the dish. The cells were observed by fluorescence microscope (BIOREVO BZ-9000, Keyence, Osaka, Japan) with the TRITC filter (excitation filter: 540/25 nm; emission filter: 605/55 nm; dichroic mirror: 565 nm) for mCherry; the YFP filter (excitation filter: 500/24 nm; emission filter: 542/27 nm; dichroic mirror: 520 nm) for Venus; the CFP filter (excitation filter: 448/20 nm; emission filter: 482/25 nm; dichroic mirror: 458 nm) for Cerulean; the DAPI filter (excitation filter: 360/40 nm; emission filter: 460/50 nm; dichroic mirror: 400 nm) for DAPI. The objective lens was a Nikon Plan Fluor 10×/0.30 and the image and data quantitative analyses of fluorescence intensity were performed as previously described [51].

### 4.6. Expression Vectors for Peptide Production in E. coli Cells

The DNAs corresponding to the HU-p53TD, the TS-p53TD, the OP-p53TD, and the GP-p53TD were synthesized as oligonucleotides (Integrated DNA Technologies) with flanking BamHI- and EcoRI-restriction enzyme sites and cloned into a modified pGEX-2T vector (Amersham, Merck, Darmstadt, Germany) with a Tobacco Etch Virus (TEV) protease cleavage site replacing the original thrombin cut site. The corresponding plasmids were transformed into *E. coli* Topp2 cells for expression.

### 4.7. Peptide Expression and Purification in E. coli Cells

For the expression, the cells were first grown overnight (16 h) in 2 L of LB with ampicillin (100 mg/L) at 37 °C, and this culture was diluted the following morning with 6 L of LB with ampicillin and induced for 4 h at 30 °C by the addition of IPTG (125 mg/L). After 4 h of induction with IPTG, the cells were harvested by centrifugation and re-suspended in lysis buffer. The cells were lysed using a French press and centrifuged at 105,000× *g* for 30 min at 4 °C. The resulting supernatant was incubated in the presence of glutathione resin (GE Healthcare) for 1 min at 4 °C. Afterwards, the supernatant was removed by centrifugation at 3000× *g* for 5 min and washed three times with TEV buffer (25 mM Na phosphate, 125 mM NaC1, 5 mM DTT). The GST-tag was removed by the overnight incubation at room temperature in the presence of 100 units of TEV protease. The solution was then centrifuged at 3000× *g* for 5 min and the supernatant was applied to an ion exchange column (buffer A: 20 mM Tris-HCl, 1 mM DTT, pH 7.2; buffer B: 20 mM Tris-HCl, 1 mM DTT, 1 M NaCl, pH 7.2). SP Sepharose or Q Sepharose column (GE Healthcare) were used. The fractions from the ion exchange column containing tag-free p53 Tet proteins were pooled and dialyzed into 20 mM Tris-HCl, 1 mM DTT, pH 7.5 for subsequent purification by gel filtration on a Superose TM 12 (GE Healthcare). The tetramer structures and their stability were measured by CD analysis as well as synthetic p53TD peptide (Figure S3).

### 4.8. Crystallization and Structure Determination

Crystals of the HU-p53TD, TS-p53TD, and OP-p53TD were grown by the vapor diffusion method at 20 °C using either 1:1 or 2:1 mixtures of peptide solution (4.0 mg/mL) and precipitant buffer. The precipitant buffer was 20% PEG 3350 with 0.2 M NH_4_NO_3_. Before the samples were flash-frozen, 24% ethylene glycol was added as a cryoprotectant. Diffraction data were collected from single crystals using the ID7B2 beamline at MacCHESS (Cornell University, Ithaca, NY, USA) or at the Canadian Light Source (CLS) (Saskatoon, SK, Canada). All data sets were processed with HKL2000. The initial phases for determining the structures were obtained by molecular replacement using the structure of the HU-p53TD as a search template. Phases were improved by iterative cycles of model building with Coot 0.9.8.93, and refinement was performed with PHENIX 1.19.2-4158. The figures were visualized using PyMOL 2.5.

### 4.9. Determination of Oxidation Status of Methionine Residues

The HU-p53TD and TS-p53TD peptides (100 μM) were first preincubated for 1h in 50 mM phosphate buffer (pH 7.5) containing 100 mM NaCl. After preincubation, hydrogen peroxide (H_2_O_2_) was added to the peptide mixture at a concentration of 100 mM to initiate the oxidation reaction. Samples were taken and analyzed by HPLC on a C8 column (4.6 × 250 mm, CAPCELL PAK C8 100, 5 mm, SHISEIDO, Tokyo, Japan) at set time intervals (0–24 h). The samples were eluted on the HPLC at a flow rate of 0.7 mL/min using buffer A (0.05% TFA in water) and buffer B (0.04% TFA in acetonitrile), using a linear gradient from 15% to 35% buffer B over 20 min to elute the peptides. Following separation on the HPLC, the different peaks were collected and digested with trypsin (0.92 mg/L, 0.16 µg) for 12 h at 35 °C. The trypsin reaction mixture was then desalted using a Macro SpinColumn™ (Harvard Apparatus, Holliston, MA, USA) and analyzed using an Ultraflextreme MALDI TOF/TOF mass spectrometer from Bruker Daltonics (Billerica, MA, USA) to verify the precise Met residues that were oxidized.

### 4.10. Statistical Analysis

Statistical analyses were performed using the Kruskal–Wallis test with Dunn’s multiple comparison post-hoc tests for transcriptional activity using GraphPad Prism (Version 9.5.1, GraphPad Software Inc., San Diego, CA, USA). For the comparisons, a *p* value of <0.05 was considered statistically significant. *** *p* < 0.0005, **** *p* < 0.0001.

## Figures and Tables

**Table 1:** Secondary structural elements present in peptides from CD analysis.

Peptides	Helix	Beta	Turn	Random
	(%)	(%)	(%)	(%)
Human (calc.) *	62.9	22.9	2.9	11.4
Human	47.3	34.4	5.6	12.7
Tree shrew	64.2	23.7	0.0	12.1
Guinea pig	47.9	23.8	0.0	28.3
Chinese hamster	50.5	33.8	13.5	2.2
Sheep	47.0	38.8	8.8	5.3
Opossum	62.0	12.7	14.7	10.7

* The contents were calculated based on the reported structure [23].

**Table 2:** Thermodynamic parameters of tetramerization domain peptides.

Peptides	*T*_m_	*ΔT*_m_	*ΔG*_u_^37°C^	*ΔΔG*_u_^37°C^
	(°C)	(°C)	(kcal/mol)	(kcal/mol)
Human	67.8 ± 0.2	-	32.4	-
Tree shrew	77.1 ± 0.2	9.3	36.4	4.0
Guinea pig	74.7 ± 0.5	6.9	35.4	3.0
Chinese hamster	69.0 ± 0.2	1.2	35.4	3.0
Sheep	75.1 ± 0.4	7.3	34.0	1.6
Opossum	78.0 ± 0.2	10.2	36.6	4.2

*T*_m_ (transition temperature) and *ΔG*_u_^37°C^ (variation in the free energy of unfolding at 37 °C) were calculated by denaturation analysis. The standard errors of fittings are indicated. *ΔΔG*_u_^37°C^ = (*ΔG*_u_^37°C^ of each species) − (*ΔG*_u_^37°C^ of Human).

**Table 3:** Molecular wight of the p53TD peptides after oxidation reaction.

	MS + 1 (324–358) *	MS + 1 (336–342) **
reduced	4231.16	980.84
mono oxi1	4247.01	996.82
mono oxi2	4247.24	980.84
di oxi	4262.91	996.82

The TS-p53TD peptide was incubated in the hydrogen peroxide. * Molecular weight of the TS-p53TD peptide after oxidation reaction. ** Molecular weight of the fragment of p53TD (residue from 336 to 342) was analyzed using MALDI-TOF MS from the fraction eluted from reverse-phase HPLC of the oxidation reaction.

## Data Availability

The atomic coordinates and structure factors have been deposited in the Protein Data Bank, Research Collaboratory for Structural Bioinformatics, Rutgers University, New Brunswick, NJ (http://www.rcsb.org/). The PDB Accession codes are: 8UQT for TS-p53TD, 8UQS for OP-p53TD and 8UQR for HU-p53TD.

## Supplementary Materials

The following supporting information can be downloaded at: https://www.mdpi.com/article/10.3390/ijms242316620/s1.

## Abbreviations

CD, circular dichroism; MALDI-TOF MS, Matrix Assisted Laser Desorption/Ionization-time of flight mass spectrometry; p53TD, tetramerization domain of p53 protein; ROS, reactive oxygen species; CH-p53TD, Chinese hamster p53 tetramerization domain; GP-p53TD, guinea pig p53 tetramerization domain; HU-p53TD, human p53 tetramerization domain; OP-p53TD, opossum p53 tetramerization domain; SH-p53TD, sheep p53 tetramerization domain; TS-p53TD, tree shrew p53 tetramerization domain; ZF-p53TD, zebrafish p53 tetramerization domain.
