# Supplementary material for: Highly Similar Tetramerization Domains from the p53 Protein of Different Mammalian Species Possess Varying Biophysical, Functional and Structural Properties
Source: Int J Mol Sci. 2023 Nov 22;24(23):16620. doi: 10.3390/ijms242316620 (PMC10706167; doi:10.3390/ijms242316620)
Supplement: Supplementary file 1 [file ijms-24-16620-s001.zip › ijms-2693880-supplementary.pdf]

Supplementary Materials

Contents:

Supplemental Table S1. Molecular weight of the mammalian p53TD
Supplemental Table S2. Data collection and refinement statistics for p53TDs
Supplemental Figure S1. Fluorescence images of the reporter assay
Supplemental Figure S2. MALDI-TOF MS spectra of the synthesized mammalian p53TD peptides
Supplemental Figure S3. CD spectra (A) and thermal denaturation curves of the synthetic and the expressed HU-p53TD peptides
Supplemental Figure S4. Overlay of the two key structural differences between the HU-p53TD and the TS-p53TD.
Supplemental Figure S5. Overlay of the two key structural differences between the HU-p53TD and the OP-p53TD.

Supplemental Table S1. Molecular weight of the mammalian p53TD

Peptides	Calculated* ($M+H^+$)	Observed**
Human	4190.7	4190.8
Tree shrew	4231.8	4233.6
Guinea pig	4245.8	4245.9
Chinese hamster	4100.7	4100.7
Sheep	4220.8	4220.9
Opossum	4305.9	4305.9

*The calculated MH^+ (average) values were calculated by the protein prospector website at UCSF.
**The observed MH^+ molecular weights of the peptides were measured using MALDI-TOF MS (linear, positive mode).

Supplemental Table S2 Data collection and refinement statistics for p53TDs

Dataset	HU-p53TD	OP-p53TD	TS-p53TD
**Data Collection**			
Beamline	ID7B2, CHESS	ID7B2, CHESS	ID7B2, CHESS
Wavelength (Å)	0.9686	1.0000	1.0000
Space group	P 21 21 21	P 21 21 21	C 2 2 2
**Unit cell parameters**			
a, b, c (Å)	30.973, 56.437, 61.656	38.275, 50.887, 60.240	62.871, 65.806, 32.511
α, β, γ (°)	90, 90, 90	90 90 90	90 90 90
Resolution range (Å)	41.63 – 1.22 (1.264 – 1.22)	32.31 – 1.35 (1.398 – 1.35)	45.46 - 1.16 (1.201 - 1.16)
No. of unique reflections	30235 (1953)	24100 (1080)	21822 (1027)
Multiplicity	5.5 (2.4)	5.6 (1.9)	9.9 (2.8)
Completeness (%)	91.77 (60.35)	90.51 (39.14)	91.70 (43.72)
R_{merge}	0.04487 (0.2466)	0.08437(.9251)	0.04206 (0.3908)
CC_{merge}	1.0 (0.959)	0.999 (0.662)	1.0 (0.84)
I/σ(I)	19.68 (2.06)	12.46 (0.51)	28.35 (1.62)
**Refinement Statistics**			
Resolution (Å)	41.63 – 1.22	32.31 – 1.35	45.46 - 1.16
Reflections (total/test)[a]	30234 (1992)	24002 (1992)	21817 (2001)
R_{work}/R_{free} (%)	0.1464/0.1764	0.1711/0.2025	0.1736/0.1915
CC_{work}	0.967 (0.937)	0.964 (0.474)	0.947 (0.891)
CC_{free}	0.967 (0.804)	0.956 (0.395)	0.942 (0.861)
**No. of atoms (excluding hydrogens)**			
Protein	1155	1107	536
Water	147	133	99
**B factors**			
Protein	16.87	21.59	16.55
Water	29.03	32.73	28.90
**Root-mean-square deviation**			
Bond length (Å)	0.022	0.011	0.009
Bond angle (°)	1.74	1.11	1.09
**Ramachandran (%)**[b]			
Favored	98.39	100.00	100.00
Outliers	0.00	0.00	0.00

Values in parentheses are for highest-resolution shell. $\mathbf{R_{sym}} = \sum \mathbf{hkl} \sum \mathbf{i}|\mathbf{I_{hkl,i}} - \langle \mathbf{Ihkl} \rangle$, where $\mathbf{I_{hkl,i}}$ is the intensity of an individual measurement of the reflection with Miller indices **hkl** and $\mathbf{I_{hkl}}$ is the mean intensity of the reflection. $\mathbf{R_{work}} = \sum \mathbf{hkl}||\mathbf{F_o}|-|\mathbf{F_c}|| / \sum \mathbf{hkl}\ |\mathbf{F_o}|$, where $|\mathbf{F_o}|$ is the observed structure-factor amplitude and $|\mathbf{F_c}|$ is the calculated structure-factor amplitude. $\mathbf{R_{free}}$ is the R factor based on at least 500 test reflections that were excluded from the refinements. CLS (Canadian Light Source) and CHESS (Cornell High Energy Synchrotron Source).

[a] Reflection for $F_o > 0$. [b] MolProbity analysis.

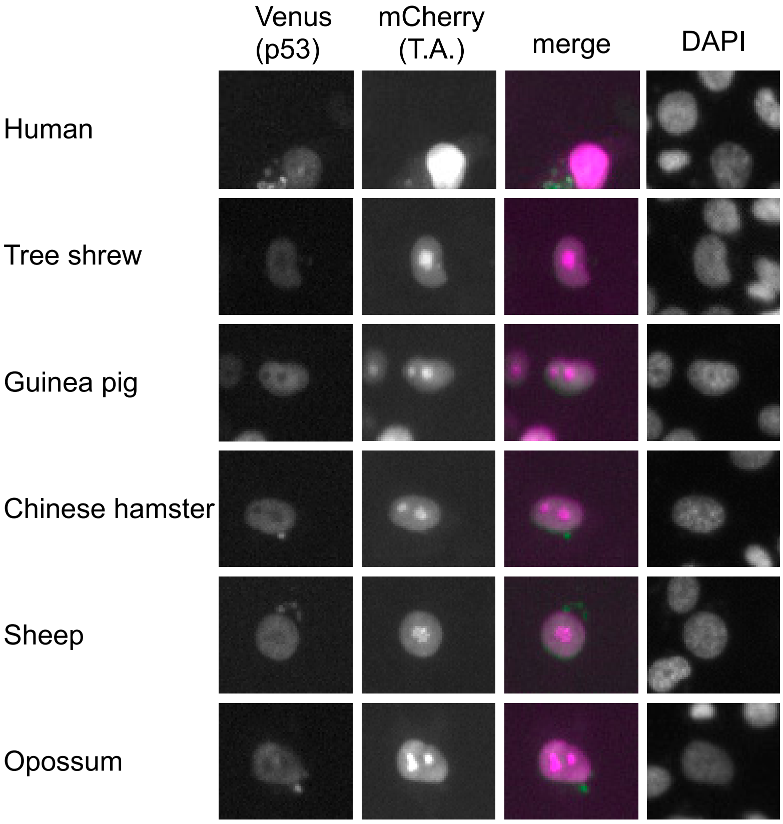

Supplemental Figure S1: Fluorescence images of the reporter assay. Chimeric p53 protein (Venus), p53-dependent transcriptional activity (mCherry), merged images of Venus (green) and mCherry (magenta), and DAPI staining were shown.

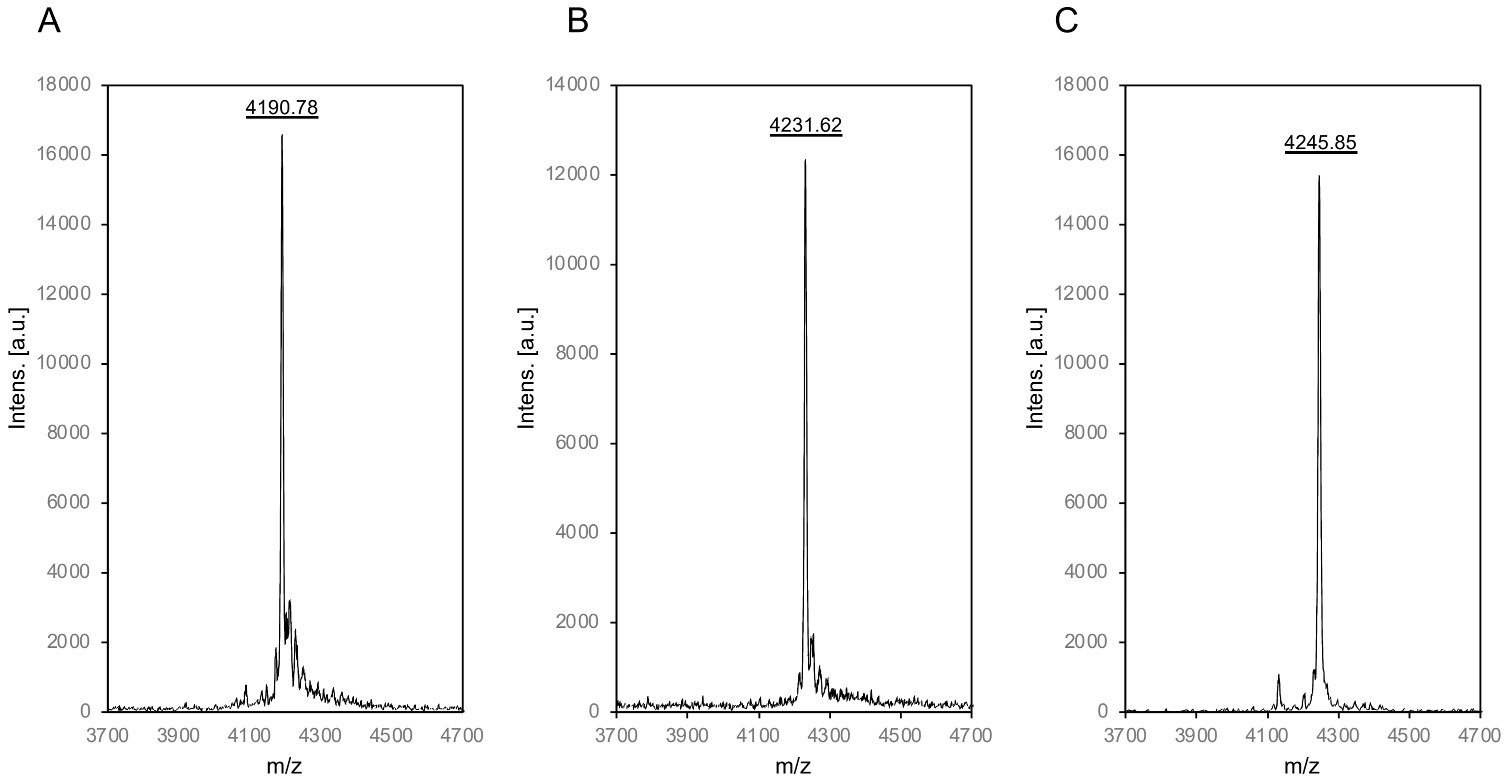

Supplemental Figure S2. MALDI-TOF MS spectra of the synthesized mammalian p53TD peptides. (A) Human, (B) Tree shrew, (C) Guinea pig, (D) Chinese hamster, (E) Sheep, (F) Opossum

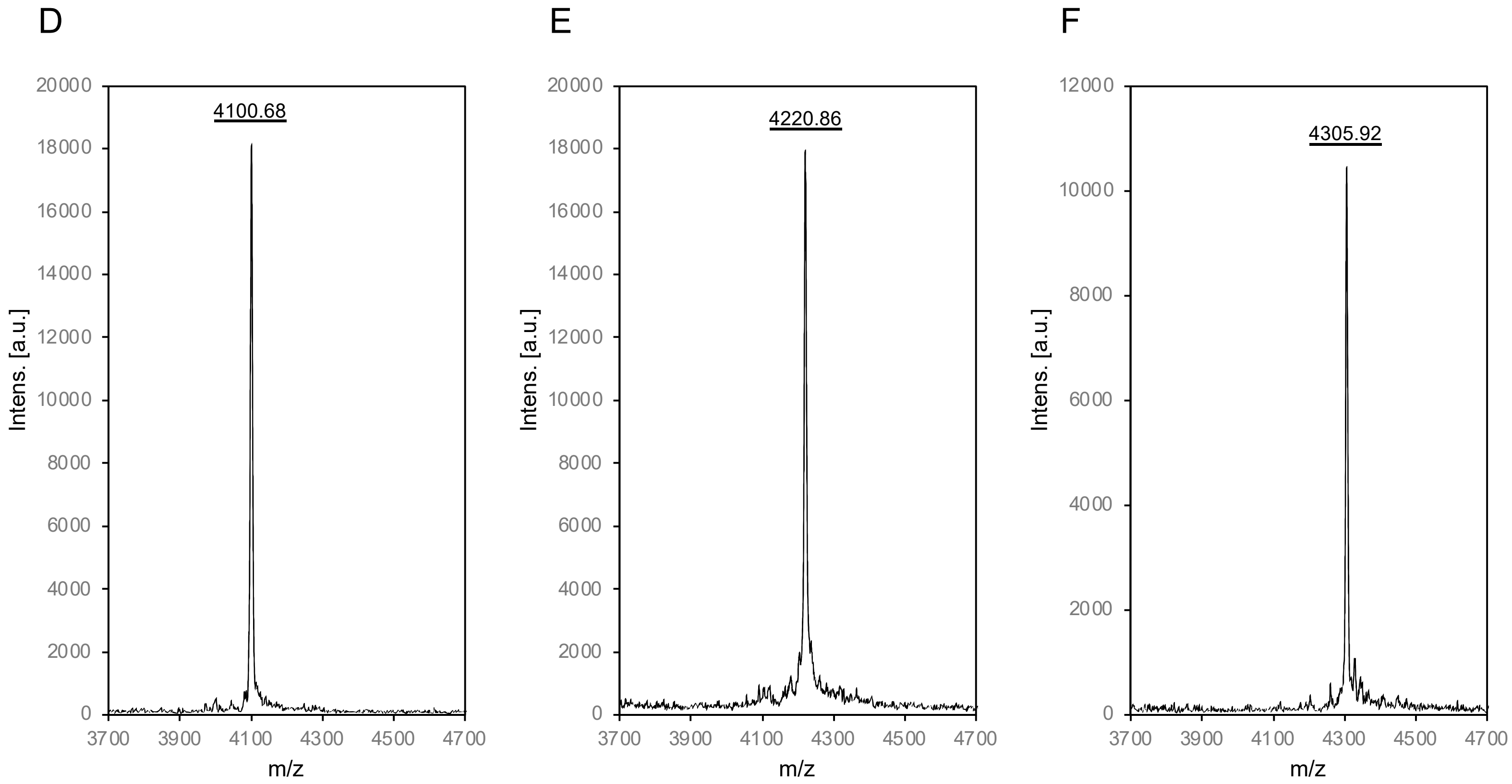

Supplemental Figure S2. MALDI-TOF MS spectra of the synthesized mammalian p53TD peptides. (A) Human, (B) Tree shrew, (C) Guinea pig, (D) Chinese hamster, (E) Sheep, (F) Opossum

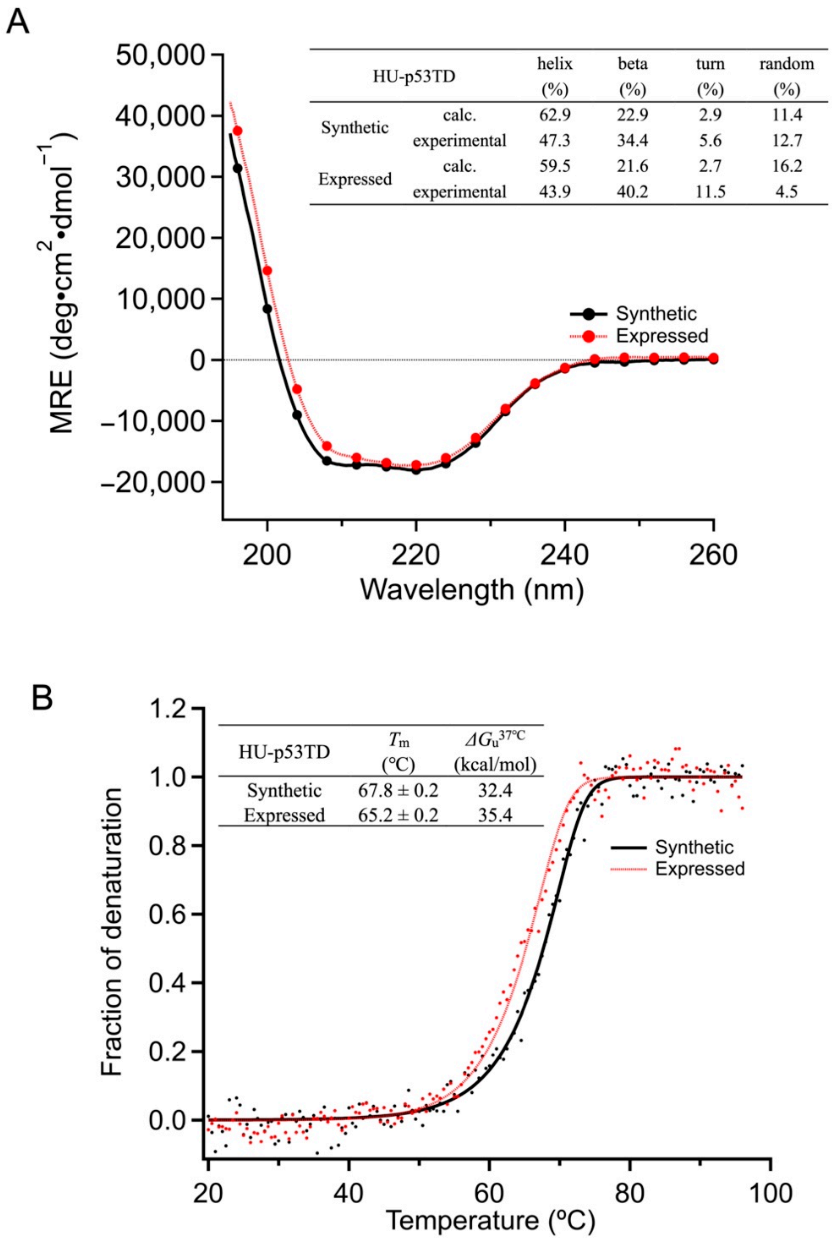

Supplemental Figure S3. CD spectra (A) and thermal denaturation curves of the synthetic and the expressed HU-p53TD peptides. (A) The secondary structure of the HU-p53TD peptides were measured by CD spectrometry at 4℃. The percentage of secondary structural elements were calculated. (B) The signals from the HU-p53TDs (synthetic and expressed in *E.coli*) were monitored by CD spectrometry at 222 nm between 4℃ and 96℃. The fraction of denaturation at each temperature was plotted to generate the denaturation curves.

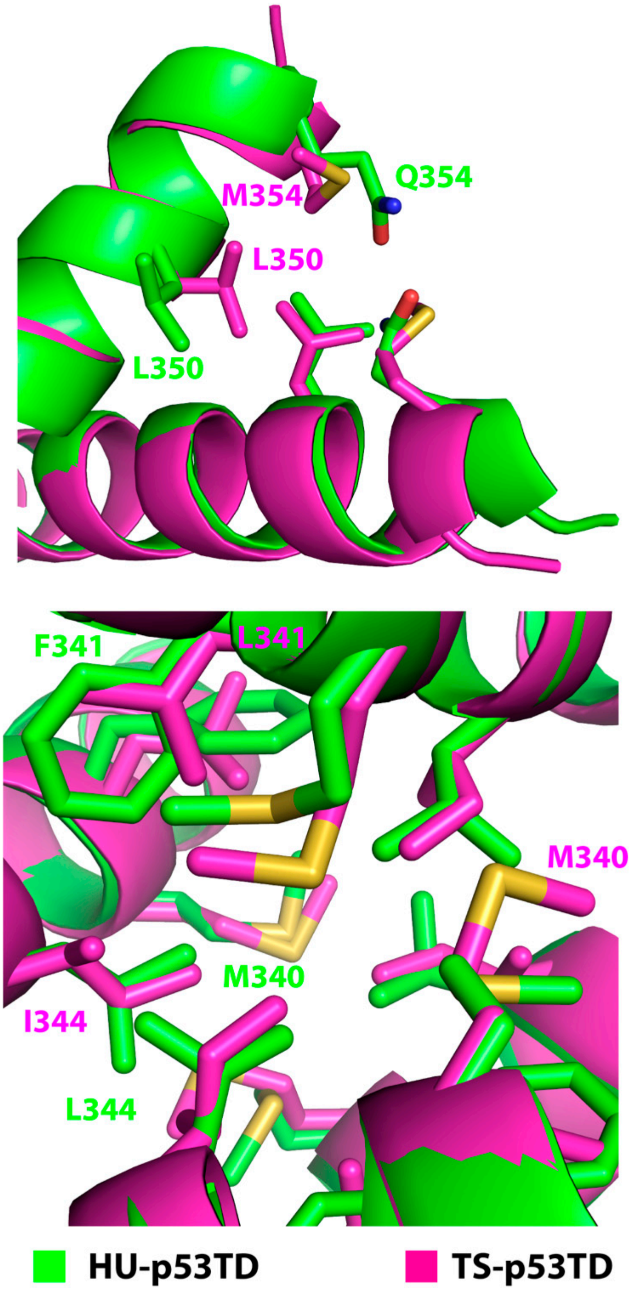

Supplemental Figure S4. Overlay of the regions containing the key structural differences between the HU-p53TD (green) and the TS-p53TD (Magenta). The upper panel shows a zoom of the C-terminal end of the α-helix highlighting the side chains of residues L350 and Q354 of HU-p53TD (green) and the corresponding L350 and M354 of TS-p53TD (magenta). The lower panel shows the zoom of the central region of the α-helix highlighting the side chains of residues M340, F341 and L344 of HU-p53TD (green) and the corresponding M340, L341 and I344 of TS-p53TD (magenta).

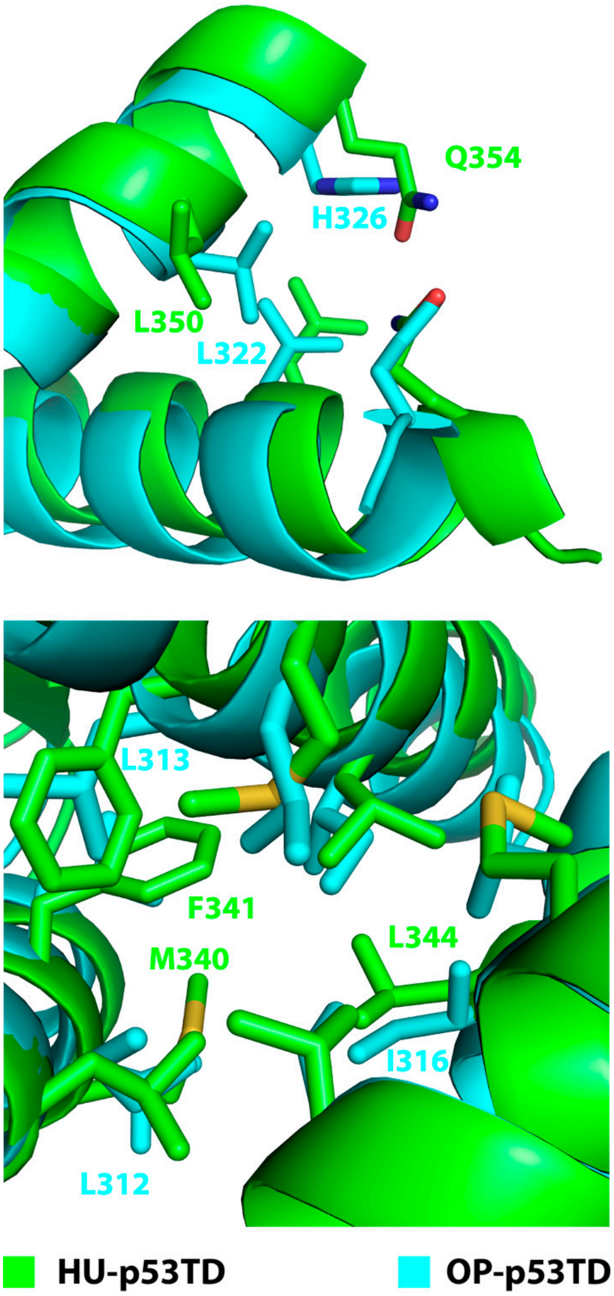

Supplemental Figure S5. Overlay of the regions containing the key structural differences between the HU-p53TD (Green) and the OP-p53TD (Aqua). The upper panel shows a zoom of the C-terminal end of the α-helix highlighting the side chains of residues L350 and Q354 of HU-p53TD (Green) and the corresponding L322 and H326 of OP-p53TD (Aqua). The lower panel shows the zoom of the central region of the α-helix highlighting the side chains of residues M340, F341 and L344 of HU-p53TD (Green) and the corresponding L312, L313 and I316 of OP-p53TD (Aqua)
